# The Case for the Anesthesiologist-Informaticist

**DOI:** 10.2196/32738

**Published:** 2022-02-28

**Authors:** Robert Lee, James Hitt, Geoffrey G Hobika, Nader D Nader

**Affiliations:** 1 Department of Anesthesiology University at Buffalo Buffalo, NY United States; 2 Department of Anesthesiology VA Western New York Healthcare System Buffalo, NY United States

**Keywords:** anesthesia, anesthesiology, AIMS, anesthesia information management systems, clinical informatics, anesthesia informatics, perioperative informatics, health information, perioperative medicine, health technology

## Abstract

Health care has been transformed by computerization, and the use of electronic health record systems has become widespread. Anesthesia information management systems are commonly used in the operating room to maintain records of anesthetic care delivery. The perioperative environment and the practice of anesthesia generate a large volume of data that may be reused to support clinical decision-making, research, and process improvement. Anesthesiologists trained in clinical informatics, referred to as informaticists or informaticians, may help implement and optimize anesthesia information management systems. They may also participate in clinical research, management of information systems, and quality improvement in the operating room or throughout a health care system. Here, we describe the specialty of clinical informatics, how anesthesiologists may obtain training in clinical informatics, and the considerations particular to the subspecialty of anesthesia informatics. Management of perioperative information systems, implementation of computerized clinical decision support systems in the perioperative environment, the role of virtual visits and remote monitoring, perioperative informatics research, perioperative process improvement, leadership, and change management are described from the perspective of the anesthesiologist-informaticist.

## Introduction

The digital evolution of health care is occurring at an accelerating rate, and clinical practice is increasingly informed by software such as electronic health records (EHRs). EHRs in the United States spread widely after the establishment of government incentive programs, and their adoption has spurred the development of information technology systems within health care including software for billing and laboratory systems. The rapid generation of large quantities of electronic forms of patient data has benefits to health care including electronic storage, easy dissemination, and expanded use in research-, clinical-, or business-related processes. The perioperative environment is data-rich, and anesthesia care generates large quantities of patient data that may potentially be captured for later secondary clinical or operational use [1,2]. Data in health care has been described as “big data,” and its increase in quantity, variety, and rapidity of data generation has defied legacy attempts at storage and use [3]. This change has necessitated the development of the discipline of biomedical informatics and the subdiscipline of clinical informatics. New techniques and tools have been created to capture, store, manipulate, and visualize data, transforming it into usable information. Eventually, artificial intelligence trained on this captured data may drive predictive analytics that inform clinical decision-making in real time in the operating room.

Anesthesiologists are at the forefront of the digital transformation of health care as it applies to the perioperative environment. Anesthesiologist-informaticists must grapple with the effective use and optimization of anesthesia information management systems (AIMS) and EHRs. Although AIMS have been shown to have several benefits around improvement in clinical documentation and reimbursement, there have been concerns around the high costs and resistance to changes in clinical work patterns [4]. Clinical informatics researchers are beginning to use the data captured in these systems to improve patient safety, quality, and care outcomes [5]. Two examples of efforts to reuse electronic data for research are the Anesthesia Quality Institute's National Anesthesia Clinical Outcomes Registry [6] and the Multicenter Perioperative Outcomes Group, which receives data from over 30 anesthesia departments AIMS [7].

In this paper, we aim to introduce clinical informatics to anesthesiologists and describe how they have the potential to make unique contributions to the field. We start by reviewing the definitions of informatics and related disciplines, as this can be a source of confusion to those outside the field. We then provide further background on the broad scope of clinical informatics as a medical specialty, and discuss options for anesthesiologists who might have interest in further training. We follow this with an overview of anesthesiology informatics and related topics including electronic anesthesia records, perioperative computerized decision support, and virtual patient care. We conclude with a brief review of perioperative informatics research and the role of the anesthesiologist-informaticist in leading and managing change in organizations, with particular focus on health care information technology as applied to the perioperative setting.

## Informatics Defined

The concept of informatics comes from the philosophy of information, where information is defined as “data plus meaning.” The term “informatics” derives from computer science and in part from information science, with some European academic departments of computer science retaining this name [8,9]. The definitions of the informatics disciplines related to health care are briefly summarized in Table 1.

**Table 1:** Definitions of informatics disciplines.

Informatics discipline	Definition
Bioinformatics	The study of information in biology, especially molecular biology, and often used to refer to data generated by genomics research [10]
Biomedical informatics	The study of information as applied to biomedical science and used to inform clinical care, medical research, and public health [11]
Clinical informatics	The medical specialty involved in the use and management of information generated by patient care, clinical research, and electronic health record systems [12]
Public health informatics	The discipline involved in managing information in public health such as vaccine registries, biosurveillance, outbreak information, and disease surveillance [13]
Medical informatics	The management of information in health care settings [14] now often used to describe nursing or dental informatics, as opposed to clinical informatics which refers to the physician-led medical specialty
Pathology informatics	An early subdiscipline of clinical informatics, focusing on laboratory information systems, analysis of pathology images, and the use of information generated in pathology practice and research [15]
Anesthesiology informatics	A subdiscipline of clinical informatics dealing with the use of information generated by anesthesia practice and the management of anesthesia information management systems.
Surgery informatics	A subdiscipline of clinical informatics dealing with the use of information generated by surgical research and practice

## Clinical Informatics

Anesthesiologists are hospital-based physicians who are trained to care for patients throughout their life span, ranging from pediatric to geriatric patients. Anesthesiologists are mainly found in the operating room, obstetrics suite, pain clinic, and the intensive care unit (ICU); however, they have significant clinical interaction with many hospital departments. They are physicians positioned to have a good overview of hospital systems because of their wide-ranging interactions with other clinical disciplines and their understanding of both medical and surgical perspectives on patient care. Anesthesiologists are focused on patient safety and quality improvement as a routine part of their work in many institutions, often working to improve perioperative processes and outcomes. For these reasons, after obtaining additional training, anesthesiologists may find themselves well suited to working in clinical informatics, either as a department-level physician champion or engaged in a system-wide informatics leadership role.

The current era of clinical informatics began with the Health Information Technology for Economic and Clinical Health (HITECH) Act in 2009, created under Title XIII of the American Recovery and Reinvestment Act (ARRA). This Obama-administration legislation established a series of incentives for the “meaningful use” of EHRs and created the Office of the National Coordinator for Health Information Technology (ONC) to lead national efforts at improving health care information technology. This need led to the widespread adoption of EHRs in the United States and renewed interest in these systems' administration and governance [16]. The HITECH legislation established meaningful use as part of a set of priorities for developing health care information technology. At first, it entailed a series of stages with incentives for using the capabilities of EHRs and later with financial penalties for not using them [17].

Interoperability and exchange of electronic patient health data between institutions remain a significant concern for the discipline of clinical informatics. Patient sovereignty over their health data has been an elusive goal. The idea of a personal health record controlled and managed by the patient [18] who would then grant access to health care providers has not been developed into an essential part of the health care system. A system of health information exchanges has provided some degree of regional portability of patient data without ownership by any participating health care institutions [19]. True interoperability has not been achieved although efforts to create standards for accessing health data from EHRs have been made. The Health Level 7 (HL7) standard Fast Healthcare Interoperability Resources (FHIR) is one such protocol for standardized messaging that can facilitate the exchange of health information through health care information exchanges or between institutions [20].

Clinical informatics is a medical specialty at the intersection of health systems science, clinical care delivery, and information technology. All informaticists, including anesthesiologist-informaticists, must understand the broad issues involved with EHR administration and regulatory reporting, data messaging, and health information exchange. Efforts at improving the use of EHRs and the data contained therein are part of the overall goals of this specialty. In addition, anesthesia informatics encompasses specialty-specific concerns and competencies related to using the informatics body of knowledge across the perioperative clinical and research environments. Anesthesia-informaticians work toward improving clinician workflows, patient safety, and care quality in the perioperative arena [21].

Informatics is not synonymous with health care information technology (HIT), and clinical informaticians are distinct from HIT professionals. Information technology is a practical discipline that refers to the operational management of enterprise computer systems, software, and resources [14]. In contrast to HIT, clinical informatics is a scientific discipline that includes theoretical and practical knowledge regarding the use of information to improve systems in health care and address the triple aim of enhancing the quality of care for populations and individual patients and improving the cost-effectiveness of care. Clinical informatics has increasingly been called on to address provider satisfaction, burnout, and the equity of care delivery [22]. Notable goals of the informatics specialty include implementation and optimization of EHRs, the reuse of EHR data, the creation of computerized decision support tools, and the maintenance of privacy and security of health care information systems.

The medical specialty of clinical informatics encompasses the clinical care of patients, a systems-based understanding of the health care environment, and an understanding of information technology. Accordingly, informaticists must have mastery of medical knowledge, informatics, the health care system, the evaluation and function of health care information systems, human factors including how clinicians interact with those systems, and how to lead and manage change in organizations. The latter includes managing teams, understanding the discipline of project management, and effective strategic and financial planning for health information systems [23].

There are several roles for physicians specializing in clinical informatics. An executive-level role, the chief medical informatics officer or chief clinical informatics officer, has been created at many hospitals and health care systems. Often reporting to the chief medical officer, chief information officer, or both, this physician bridges between the clinical concerns of the medical staff and the technical health care information technology needs in an organization. She may provide clinical oversight for the electronic health record and delegate requests for informatics resources for clinical, research, or business intelligence purposes [24]. A second typical physician role is the physician champion, who is often involved with EHR implementation, ongoing optimization, and provider education. Leadership and change management are core competencies for clinical informaticists. Both chief medical informatics officers and physician champions take part in the organizational change management of EHR use and related clinical workflows [25]. Clinical informatics competency has become essential for other physicians in leadership roles ranging from department chairpersons, medical directors, clinical quality officers, and those involved with the administration of value-based care. Clinical informaticists may intersect with various careers across clinical operations in health care systems, clinical or basic biomedical research, or the industry of creating health care information technology tools.

## Informatics Training Options

As clinical informatics has now become part of the medical school curriculum and residency training clinical competencies, there are several options for practicing anesthesiologists who would like to obtain additional training. The American Medical Informatics Association (AMIA) offers an online continuing medical education course called the “AMIA 10 x 10”, which is equivalent to an introductory graduate course in clinical informatics. The original intent of this course was to train 10,000 individual clinicians in informatics by 2010 [26]. The 10 x 10 online course is a good first step for practicing anesthesiologists who would like to learn more about clinical informatics with minimal time commitment.

At many institutions, master's degree–level training is available in biomedical informatics for those wishing to specialize in clinical informatics. The AMIA has defined a curriculum for core competencies for graduate education in biomedical informatics. These include fundamental skills around formulating and solving scientific problems, familiarity with scientific issues in biomedicine and population health, theories and methodologies particular to biomedical informatics, and technology-based skills and approaches [27]. Interested anesthesiologists may elect to pursue a master’s degree online or on a part-time basis while they continue to practice. This may appeal to anesthesiologists who plan to integrate perioperative informatics research with ongoing clinical practice. Some physicians may elect to continue their education and pursue a doctorate in philosophy (PhD) in biomedical informatics. This pathway, while not as common for trained physicians, has been undertaken by some who are planning to devote a significant portion of their career to informatics research. The National Libraries of Medicine (NLM) has been the primary supporter of graduate and postgraduate training in biomedical informatics since the 1970s and has provided graduate and postdoctoral fellowships for training in informatics [28].

The formalization of clinical informatics as a medical specialty started in 2007 when the American Medical Informatics Association became a full member of the Council of Medical Specialty Societies, a group of organizations that offer board certification through the American Board of Medical Specialties (ABMS) [29]. A core content outline was developed [23], and fellowship training requirements were described [30]. Permission was granted in 2010 for ABMS board certification administered jointly by the American Board of Pathology and the American Board of Preventative Medicine [31]. The first clinical informatics board certification cohort was in 2013. From 2013 through 2018, there were 1983 certifications issued, with 71 anesthesiologists receiving board certification. In terms of surgical specialists, during the same timeframe, 44 certificates were issued to general surgeons, 39 to obstetricians and gynecologists, 14 to ophthalmologists, 12 to head and neck surgeons, 10 to urologists, 5 to orthopedic surgeons, 3 to thoracic surgeons, 2 to colorectal surgeons, 2 to neurosurgeons, and 2 to plastic surgeons [32].

There is currently a practice pathway to board certification open to anesthesiologists and other physicians, requiring 25% effort dedicated to clinical informatics in 3 of the 5 years prior to application to take the board examination. The practice pathway to board certification will end after 2022, and after that time, interested physicians must complete a 2-year Accreditation Council of Graduate Medical Education (ACGME)–accredited fellowship in clinical informatics to attain eligibility for ABMS board certification in clinical informatics [32]. Physicians from any specialty may enter fellowship training. The fellowship programs are administered through a clinical department that may be from any primary medical specialty.

Finally, the AMIA Health Informatics Certification (AHIC) is open to physicians and nonphysicians who have completed an informatics graduate degree (master’s or doctorate) and who have qualifying work experience in health care informatics. Anesthesiologists or other physicians interested in certification via the AHIC pathway must have spent at least 20% of their work time in health informatics during 8 of the preceding 10 years, or 50% or more time in health informatics work during 6 of the past 8 years. Qualifying candidates must pass a multiple-choice examination to earn their certification [33]. We expect that after the close of the practice pathway to ACGME board certification at the end of 2022, certification via AHIC will appeal to anesthesiologists who are working in clinical informatics on a part-time or full-time basis.

## Anesthesiology Informatics

**Figure 1:**
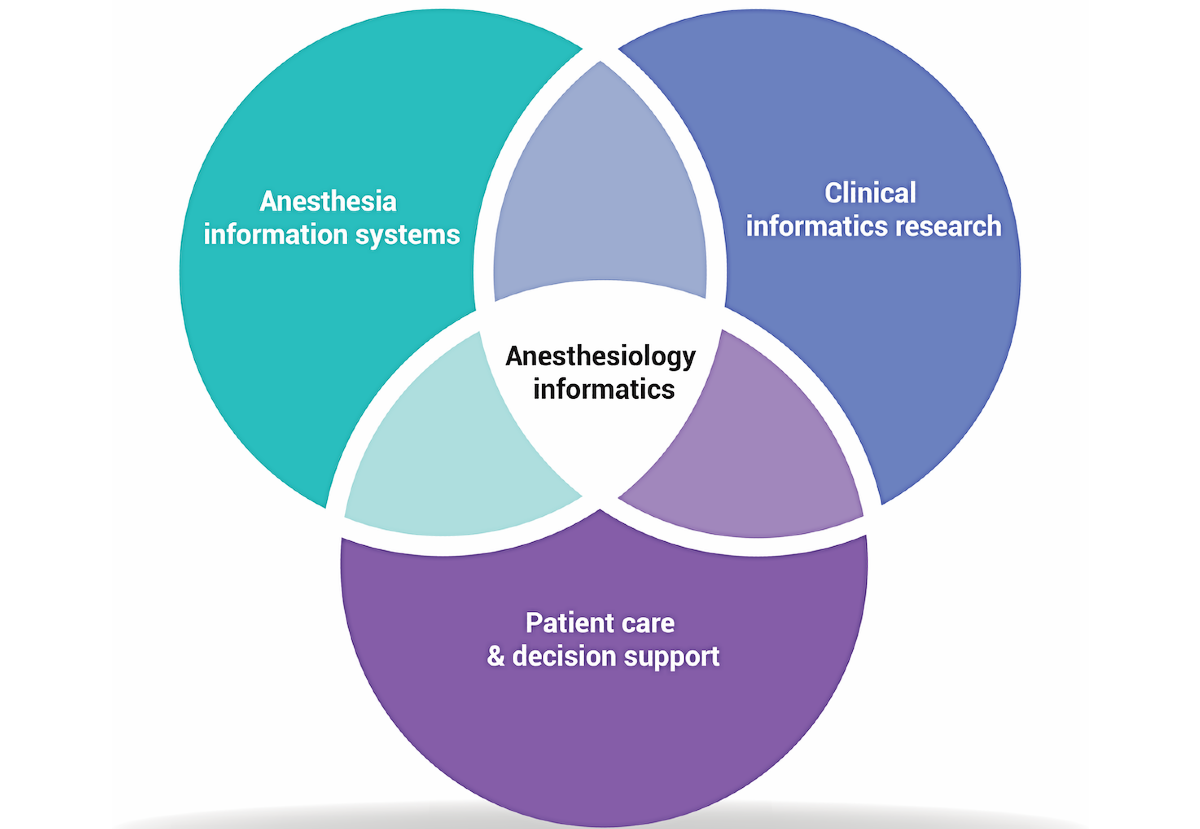
Anesthesiology informatics.

Anesthesiology informatics involves the intersection of medical care for surgical and procedural patients, perioperative clinical decision support, the management of anesthesia information systems, and anesthesia informatics research (Figure 1). Anesthesiologist-informaticists often act as physician champions for their anesthesia information management systems that capture patient vital signs, ventilator parameters, and medication administrations to form a complete and accurate medical record of anesthetic care. [21] Pain Medicine subspecialist anesthesiologists routinely use enterprise EHRs for patient care and may be involved in developing or using clinical decision support systems (CDSS) focused on the safe treatment of patients with acute or chronic pain [34,35]. Colleagues focused on resident and medical student education may run advanced, computerized simulation centers [36]. Anesthesiologist-informaticists involved in quality assurance, patient safety, or research may be interested in the secondary use of clinical data to measure patient outcomes across or between clinical populations. As of 2016, most board-certified anesthesiologist-informaticists had additional informatics duties outside of their clinical department while working as anesthesiologists. The majority did not have formal training in informatics [37].

A significant part of the effort of anesthesiologist-informaticists goes toward the implementation, optimization, and management of anesthesia information management systems (AIMS) which are the EHR components that keep the record of the intraoperative course of patients undergoing anesthesia. The AIMS may solely deal with tracking intraoperative vital signs, medication administration, and intraoperative events or may be more complex and encompass preoperative evaluation and postoperative care documentation. In turn, these systems may supply data for advanced analytics and visualization for clinical or research purposes [4,38].

The Society for Technology in Anesthesia (STA) is an international organization for anesthesiologists who have a particular interest in the technologies used in the operating room and the perioperative environment. The STA was founded in 1988 to improve the quality of patient care by improving technology and may serve as a home organization for anesthesiologists with a special interest in clinical informatics [39]. The STA mission encompasses all technology used in anesthesia practice and including but not limited to the digital technologies of interest to clinical informaticists. Outside of the STA, in an effort to improve the dissemination of knowledge in the field, an open-access journal explicitly dedicated to perioperative informatics, *JMIR Perioperative Medicine*, was founded in 2018.

## Management of Perioperative Information Systems

The Anesthesia Patient Safety Foundation (APSF) was founded in 1985 with the purpose of improving patient safety throughout the entire perioperative period. This process of renewed emphasis on safety in anesthesia led to many critical monitoring advances, such as widespread intraoperative use of pulse oximetry and continuous capnography. The APSF, as an industry leading organization in patient safety, determined that, to take the next step in improving the quality and safety of anesthesia care, the profession had to embrace the importance of clinical informatics and automated perianesthetic data management. This would enable anesthesiologists to pinpoint the problems responsible for preventable anesthetic morbidity and mortality [40].

Information systems used by anesthesiologists to obtain historical and laboratory information about patients include the hospital EHRs and laboratory information systems. Health information exchanges may be used to obtain history, imaging, or lab data from outside facilities. Anesthesia information management systems are used for the documentation of the pre- and intraoperative portions of patient care. Subspecialty colleagues may use specialized electronic record systems or decision support systems in the practice of critical care medicine [41] or pain medicine. Additional components to perioperative information systems are used to manage operating room staffing, patient flow, and case scheduling. [42]. Core competencies of clinical informaticians in the perioperative environment include understanding these systems and leading efforts at system implementation and ongoing optimization.

Anesthesiologist-informaticists who manage these information systems must understand the regulations and ethical considerations around patient privacy and data security. These concerns range from safeguarding patients by understanding the regulations enacted in 1996 by the HIPAA (Health Insurance Portability and Accountability Act) and the modifications from the 2013 Final Omnibus Rule, which added rules regarding breach notification. Anesthesiologist-informaticists may be champions for secure messaging in the hospital and operating room, using secure text or voice communications.

The oversight of remote monitoring systems for the operating room or ICU may fall under the purview of critical care anesthesiologists. These systems must be designed safely, with emphasis on an intuitive user experience and clear workflow to facilitate safe and effective patient care [43]. These ICU telehealth systems lend themselves to large-scale research by collecting data across clinical sites [44]. Dashboards for displaying information about the status of patients in the operating room or ICU have similar design and deployment considerations. These tools for data visualization can be used for practical purposes like improving turnover time in the operating room.

Tracking perioperative patient flow using radiofrequency identification for real-time location tracking is also becoming part of the informaticist's competencies. There has been an interest in using these technologies to track equipment locations in the hospital and the perioperative environment [45] For example, video laryngoscopes or ultrasound machines used for the practice of regional anesthesia could be tracked inside the operating room and supporting areas.

## Clinical Decision Support in the Perioperative Environment

A major goal of informatics as a specialty has been to provide computerized clinical decision support to clinicians at the point of care. Quality clinical decision support has been characterized by the “five rights,” which involves delivering accurate information, to the right person, in the proper format, through the right channels, and at the right time in the clinical workflow [46]. Examples of essential clinical decision support include order sets, such as postoperative order sets for use in the postanesthesia care unit that provide a previously determined selection of medications, using safe and reasonable dosage ranges. Computerized provider order entry is another basic form of decision support. Limitations on dosing can help prevent error, and automated checks against patient allergies and drug-drug interactions may occur. This may be especially helpful to improving medication safety in the operating room, as anesthesiologists order, compound, and administer medications directly in the operating room without a second clinician or nurse checking their work. Clinical decision support systems in the operating room as part of the AIMS may alert providers to allergies, medication interactions, or the need to treat alterations in patient hemodynamics. They can also give reminders as to the timely and appropriate administration of perioperative antibiotics and beta-blockers [47].

Alarm fatigue is well-known to anesthesiologists from the operating room [48] and is a topic relevant to the design and implementation of CDSS. When alarms are too frequent or meaningless, providers pay less attention to the signals, with the potential result that important, critical information may be missed. This is not only an issue in the operating room and the critical care environment, but has relevance to the design of clinical decision support pop-up messages in the AIMS or EHRs for alerting providers of an order with potential drug interactions or other patient safety issues [49]. Informaticians must design and manage CDSS to maintain potential safety benefits while minimizing clinician alarm fatigue and maintaining vigilance when presented with alerts.

## Virtual Visits

There has been a surge in interest in the use of telehealth for patient visits during the COVID-19 pandemic to decrease patient throughput inside health care facilities. Indeed, widespread implementation of telehealth was undoubtedly a positive consequence of the pandemic and is here to stay. The idea of a medical virtualist as a distinct specialty for the provision of telehealth has been proposed [50]. Clinical informaticists consider telehealth systems as part of their expertise in health care information systems. Telemedicine systems may improve access to surgical specialists and decrease the time patients wait to be seen. Telehealth systems may facilitate postoperative follow-up by reducing patient travel time [51]. For anesthesiologist-informaticists, the use of virtual visits may include making preoperative and postoperative patient evaluation available without requiring patients to travel to the health care facility before or after their surgery [52].

Remote monitoring may be considered a form of virtual care. This has become commonplace in the ICU, with remote monitoring by intensivists providing additional patient safety [53]. Anesthesiologists using a team-based approach to care may monitor a patient’s vital signs and anesthesia care information remotely from outside the operating room. Some facilities include video capability to observe the patient during anesthesia and surgery remotely [54].

Other forms of virtual interaction with the patient may include the asynchronous use of patient portals where patients may message providers, review their medical records, or request medication refills [55]. Pain medicine subspecialist anesthesiologists make extensive use of these patient portals to facilitate communication with patients. The Open Notes movement has made it so that many institutions provide full and immediate access to the patient of their medical records in an online format, which has led to improved communication between patients and clinicians [56].

## Perioperative Informatics Research

Anesthesiologist-informaticists may be involved in clinical informatics research. The collection of patient information by EHRs and AIMS allows for data warehousing and secondary data reuse to inform extensive database studies. This can facilitate perioperative outcomes research through the use of data from large numbers of patients and quality improvement programs. It may become possible to solve challenging-to-answer clinical research questions that require large amounts of retrospective patient data for achieving adequate statistical power [57]. Two well-known examples of large anesthesia data aggregators are the Anesthesia Quality Institute's National Anesthesia Clinical Outcomes Registry [6] and the Multicenter Perioperative Outcomes Group, a data warehouse fed from multiple anesthesia information management systems [7]. The American College of Surgeons National Surgical Quality Improvement Program (ACS NSQIP) is an example of an extensive collection of surgical outcomes data.

There are persistent issues with the quality of data obtained from AIMS, especially missing or duplicated values [58]. Issues related to poor documentation quality and missing data are also found in EHR databases and present one of the major challenges that informaticists face when doing research. As techniques have been developed to work around data quality issues, the future design of EHRs and clinical databases may better address data quality concerns.

Further directions in informatics research include machine learning and natural language processing to obtain information from the accessible text portions of EHR data [59]. The improvement of how data is indexed from EHRs using ontologic approaches to make data computable and interchangeable between different electronic records is ongoing.

## Perioperative Process Improvement, Leadership, and Change Management

Informaticists use health care systems knowledge and health information technology knowledge in an increasingly computerized clinical care setting to lead and affect change and improvements in clinical environments. It has been theorized that failures in the implementation of computerized clinical information systems have behavioral causes. For this reason, informaticists must develop leadership and change management competencies [60]. They may also be called upon to use their expertise to inform health care policy decisions.

Leadership efforts by anesthesiologist-informaticists include quality improvement in the perioperative environment. Quality improvement or quality assurance using EHR and AIMS data to compare patient outcomes against standard quality measures has become an important part of ensuring patient safety and reporting to insurance payors or regulatory authorities.

Burnout among anesthesiologists and surgical specialists has been reported to be at significant levels [61]. The computerization of clinical work—and specifically EHRs that are difficult to use—has been tied to decreased provider satisfaction and has been partly blamed for the trend toward clinician burnout [62]. Informaticists have a role in preventing and mitigating burnout by optimizing EHR systems to improve clinician workflows.

Informaticists may help perioperative clinicians with improving clinical documentation, including preoperative patient evaluations. They also teach providers how to document clinical care efficiently and effectively, with emphasis given to problematic issues like copying and pasting text from old records. Changing rules around clinical documentation may be monitored by anesthesiologist-informaticists who can then, in turn, disseminate information to colleagues and assist with changes in EHR templates and clinical workflows. Anesthesiologist-informaticists may be called on to map provider workflows in the operating room environment with an eye toward process improvement.

Ransomware attacks against health care systems and data breaches involving patient medical records are now frequently reported, and trained clinical informaticists can help lead efforts directed at risk mitigation and emergency response. Disaster planning for events such as the COVID-19 pandemic and other as-yet-unforeseen events requires attention to informatics infrastructure.

Informing clinicians regarding the use of social media in such a way as to ensure patient privacy falls under the responsibilities of the clinical informaticist. Anesthesiologists may use social media for sharing and disseminating information from the medical literature. Many anesthesia conferences including the American Society of Anesthesiologists’ Annual Meeting and the New York State Society of Anesthesiologists’ Post-Graduate Assembly now commonly use social media to inform attendees and have transmitted hashtags for use in attendee social media posts.

## Conclusions

Anesthesiologist-informaticists have value in leading implementation and ongoing optimization efforts for anesthesia information management systems. They may improve clinical decision support systems for perioperative care or improve remote monitoring for patients in the operating room.

Anesthesiologist-informaticists will face a growing number of challenges in the future - acceleration in the use of perioperative health care information technology continues. There is increased volume and rapidity of data collection in the operating room environment. This will present challenges in how to best warehouse and transform this data for secondary use in research, clinical decision support, and informing business decisions in health care organizations. Integrating consumer health data into the preoperative EHR will present challenges around how to best use these data for preoperative evaluation and to improve patient outcomes, and require clinician oversight. As improving machine learning techniques make artificial intelligence a routine part of predictive analytics and advanced clinical decision support in the operating room, anesthesiologist-informaticists will be called on to ensure safe, effective, and ethical use of these new technologies.

In this paper, we have described the discipline of clinical informatics, presented options for anesthesiologists who would like to obtain further informatics training, and attempted to make the case that the anesthesiologist-informaticist can make significant contributions to meeting the challenges of the emerging field of perioperative informatics.

## Abbreviations

ABMS: American Board of Medical Specialties
ACGME: Accreditation Council for Graduate Medical Education
ACS NSQIP: American College of Surgeons National Surgical Quality Improvement Program
AIMS: anesthesia information management system
AMIA: American Medical Informatics Association
APSF: Anesthesia Patient Safety Foundation
ARRA: American Recovery and Reinvestment Act
CDSS: Clinical Decision Support System
EHR: electronic health record
FHIR: Fast Healthcare Interoperability Resources
HIPAA: Health Insurance Portability and Accountability Act
HITECH: Health Information Technology for Economic and Clinical Health
HL7: Health Level 7
ICU: intensive care unit
NLM: National Libraries of Medicine
ONC: Office of the National Coordinator
STA: Society for Technology in Anesthesia
